# The differential expression profiles of miRNA in serum-derived exosomes and its potential role in age-related hearing loss

**DOI:** 10.3389/fnagi.2025.1694514

**Published:** 2026-01-14

**Authors:** Juhong Zhang, Haizhu Ma, Jing Ke, Ziyi Tang, Zhiji Chen, Guijun Yang, Li Yang, Jialin Guo, Xiaoqi Yan, Changxiu Peng, Kaiye Wang, Xiyao Chen, Shaojing Kuang, Wei Yuan

**Affiliations:** 1 Department of Otorhinolaryngology Head and Neck Surgery, Chongqing General Hospital, Chongqing University, Chongqing, China; 2 CQMPA Key Laboratory for Development and Evaluation of Innovative Biological Products, Chongqing, China

**Keywords:** age-related hearing loss, exosomes, miRNA transcriptome sequencing, miRNA, hearing

## Abstract

**Objectives:**

This study aimed to characterize serum exosomal miRNA profiles from patients with age-related hearing loss (ARHL) to identify key pathogenesis-related miRNAs for ARHL.

**Methods:**

Peripheral venous blood samples were collected from patients with ARHL and elderly controls, and exosomes were isolated from serum of each subject. Then, the isolated exosomes were systematically identified by nanoparticle tracking analysis (NTA), transmission electron microscopy (TEM) and western blot. Subsequently, the isolated exosomes were submitted for miRNA sequencing and a series of bioinformatics analysis. Ultimately, four key DE-miRNAs, namely hsa-miR-100-5p, hsa-miR-23b-3p, hsa-miR-373-3p, and hsa-miR-27b-3p, were verified using quantitative real-time polymerase chain reaction (RT-qPCR).

**Results:**

NTA, TEM and western blot confirmed exosomes were successfully isolated. After sequencing, 22 differential expressed miRNAs (6 up-regulation and 16 down-regulation) were identified between the exosomes from ARHL and controls, and then collectively identified 17,451 predicted target genes and 15,863 experimentally validated target genes. Gene Ontology enrichment analysis revealed that the target genes were significantly associated with “regulation of neuron projection development,” “sensory system development,” “proteasome-mediated ubiquitin-dependent protein catabolism,” and “ubiquitin-like protein ligase binding.” Kyoto Encyclopedia of Genes and Genomes (KEGG) showed the target genes were significantly enriched in “PI3K-Akt signaling pathway,” “MAPK signaling pathway,” “cellular senescence,” “autophagy,” “mTOR signaling pathway,” “ubiquitin-mediated proteolysis,” and “signaling pathways regulating stem cell pluripotency.” Additionally, the Reactome analysis highlighted the involvement of “MAPK family signaling cascades,” “negative regulation of the PI3K/AKT network,” and “antigen processing: ubiquitination and proteasome degradation.” Disease Ontology further demonstrated significant enrichment of target genes in neurological disorders. RT-qPCR showed hsa-miR-100-5p and hsa-miR-23b-3p exhibited markedly decreased levels, while hsa-miR-373-3p and hsa-miR-27b-3p were significantly up-regulated in ARHL, which were consistent with sequencing results, confirming a high relatively reliability of the sequencing results.

**Conclusion:**

Ubiquitination modification, autophagy process, cellular senescence and nervous system regulation may jointly contribute to the core molecular mechanism of ARHL. The hsa-miR-100-5p, hsa-miR-23b-3p, hsa-miR-373-3p, and hsa-miR-27b-3p may preliminarily act as key regulatory factors to participate in the pathophysiological process of ARHL, providing exploratory evidence for their potential application value as molecular markers.

## 1 Introduction

Age-related hearing loss (ARHL), also known as presbycusis, is a disease associated with aging that concurrently impacts both the peripheral auditory system and the central auditory pathways (Mussoi and Brown, 2019). This condition exhibits a high prevalence globally. By 2024, the number of people aged 65 and above will exceed 240 million, accounting for approximately 16.5% of the total population, among which two-thirds have hearing loss (Ninoyu and Friedman, 2024). ARHL is not only characterized by high-frequency hearing loss and impaired speech recognition ability (Dubno et al., 2008), but is also closely related to cognitive decline and increased risk of dementia. Timely hearing aid intervention can effectively delay the deterioration of cognitive function (Lee, 2015). With the acceleration of the global aging population, hearing impairment characterized by both peripheral and central lesions has transcended a mere sensory function defect. It is progressively emerging as a significant public health concern that exacerbates the burden on an aging society. Consequently, a thorough investigation into its early warning mechanisms and effective intervention strategies is of considerable social value and practical importance.

As a degenerative disease with a complex pathogenesis and limited clinical treatment options, ARHL still lacks reliable biomarkers for early diagnosis and intervention at present. Although emerging evidence suggests that Apela/Toddler peptide may serve as a potential biomarker for age-related disorders (Monastero et al., 2023), and the auditory brainstem gap response has been proposed as a novel physiological indicator for ARHL (Williamson et al., 2015), these findings remain preliminary. With the accelerating global aging population, the prevalence of ARHL is projected to rise substantially, posing dual challenges to healthcare systems: On the one hand, there is an urgent need to develop functional and molecular biological markers capable of identifying preclinical reversible lesions. On the other hand, effective early warning intervention programs need to be established to address this increasingly severe public health issue.

MicroRNA (miRNA) is a type of endogenous small non-coding RNA approximately 20–24 nucleotides in length. It regulates gene expression at the post-transcriptional level by binding to the 3′ untranslated region (3′-UTR) of the target gene. In the cochlea of aged C57BL/6J mice, activated miR-34a impairs autophagic flux through ATG9A suppression, ultimately promoting cochlear cell death (Pang et al., 2017). Similarly, miR-204-5p regulates SIRT1 to promote endoplasmic reticulum stress-induced apoptosis of inner ear hair cells in aged mice (Hu et al., 2024). Our previous work demonstrated that miR-130b-3p delays cellular senescence by enhancing autophagy in sensory cells via targeting PPARγ (Zhang et al., 2024). These findings collectively underscore the pivotal regulatory functions of miRNAs in ARHL occurrence.

In recent years, exosome secretion and its role in diseases have received increasing attention. Exosomes are membrane vesicles with a diameter of 40–100 nm, encapsulated by a lipid bilayer. They can secrete circulating miRNA, and the miRNAs in exosomes are more stable during the circulation process, thus having greater potential value as biomarkers (Nik Mohamed Kamal and Shahidan, 2020; Redis et al., 2012). In 2018, Wong et al successfully extracted exosomes from the inner ear of Wistar rats for the first time (Wong et al., 2018). Research has found that exosomes derived from neural progenitor cells prevent hearing loss caused by ischemia-reperfusion injury in mice by inhibiting cochlear hair cell inflammation (Hao et al., 2022). Mesenchymal stem cell-derived exosomes increase the autophagic activity of hair cells, promote cell survival, reduce mitochondrial oxidative stress levels and the apoptosis rate of hair cells, thereby improving neomycin-induced ototoxicity (Liu et al., 2024b). However, there are relatively few studies on the relationship between exosomes and ARHL.

This study employed high-throughput sequencing technology to systematically analyze the expression profiles of miRNAs in serum exosomes of patients with ARHL, aiming to screen out key miRNAs closely related to the pathogenesis of ARHL and evaluate their potential value as biomarkers for disease diagnosis. The findings will provide novel insights into the molecular mechanisms underlying ARHL and may yield promising molecular targets for early clinical diagnosis and intervention.

## 2 Materials and methods

### 2.1 Ethical review

This study was approved by the Medical Ethics Committee of Chongqing General Hospital (approval no. KY S2023-044-01). Prior to sample collection, written informed consent was obtained from all participants, including 6 ARHL patients and 6 age-matched healthy controls recruited for this study. The clinical information of enrolled participants is summarized in Table 1 and Supplementary Figures 1, 2.

**TABLE 1:** The general condition and hearing information of the subjects.

Group	No	Sex	Age	Pure tone hearing, dB HL (right)						Pure tone hearing, dB HL (left)						Aim
				250 Hz	500 Hz	1,000 Hz	2,000 Hz	4,000 Hz	8,000 Hz	250 Hz	500 Hz	1,000 Hz	2,000 Hz	4,000 Hz	8,000 Hz	
ARHL	1	Male	60	40	45	55	45	50	60	40	45	55	50	55	70	Sequencing
ARHL	2	Male	66	45	40	45	45	55	70	40	45	45	50	60	65	
ARHL	3	Female	65	35	35	40	45	50	65	35	35	45	45	55	65	
ARHL	4	Male	76	50	45	30	25	75	65	60	55	30	30	80	70	RT-qPCR
ARHL	5	Female	74	50	50	55	55	60	75	50	50	55	60	75	70	
ARHL	6	Female	76	70	80	80	80	75	80	50	60	60	65	60	95	
Control	1	Female	80	10	5	10	10	20	20	5	10	15	10	20	25	Sequencing
Control	2	Male	62	10	10	10	15	25	25	5	15	10	15	25	20	
Control	3	Male	62	5	5	20	20	20	25	5	10	20	20	25	25	
Control	4	Female	78	30	25	20	25	30	45	35	30	20	20	35	40	RT-qPCR
Control	5	Female	73	25	20	25	20	25	35	20	20	20	15	20	30	
Control	6	Male	68	20	20	10	5	10	10	20	20	10	5	10	5	

### 2.2 Collection of subjects

The study participants were divided into two groups: ARHL group and an elderly control group. According to the World Health Organization (WHO) diagnostic criteria for ARHL (World Health Organization, 2021), pure-tone audiometry was conducted by a certified audiologist in a professional soundproof booth to ensure the accuracy of the hearing thresholds. Participants in the ARHL group were required to have a mean hearing threshold > 35 dB HL at four frequencies (0.5, 1, 2, and 4 kHz), while those in the control group were required to maintain thresholds < 20 dB HL at the same frequencies.

Inclusion criteria: (1) over 60 years old, (2) be capable of completing basic daily living activities, such as taking a bath, dressing, grooming and using the toilet; And the ability to walk independently for 400 m, (3) right-handed, (4) there was no previous history of ear trauma or surgery, and no other cranial nerve injuries except for cranial nerve VIII, (5) have not used a hearing aid either currently or before, (6) sign the informed consent form and be willing to participate in the research.

Exclusion criteria: (1) Meniere’s disease, herpes zoster oticus, noise-induced deafness, exposure to ototoxic drugs, conductive hearing loss, and other ear diseases with known causes. (2) Meningitis, cognitive impairment, metabolic diseases, cardiovascular diseases, autoimmune diseases. (3) Previous or current mental or neurological disorders, addiction to psychoactive substances or sedatives. (4) History of head trauma.

### 2.3 Serum sampling

The peripheral venous blood of six ARHL patients and six elderly controls were collected, and the serum was obtained by collecting supernatant after centrifugation at 1,900 × g for 10 min, followed by 13,000 × g for 2 min at 4 °C. Among them, three of them were used for sequencing, and the remaining three samples were employed for expression validation using real-time quantification PCR (RT-qPCR) (Table 1).

### 2.4 Isolation of serum-derived exosomes

Exosomes were isolated from serum of ARHL patients and healthy controls at 4°C using the ultra-centrifugation method. Briefly, the collected serum samples were thawed on ice, and then subjected to sequential centrifugation: initial clarification at 500 × g for 5 min, followed by transfer of the supernatant to fresh 50 mL tubes for centrifugation at 2,000 × g for 30 min. The resulting supernatant was further processed by centrifugation at 10,000 × g for 60 min. The clarified supernatant was then filtered through 0.22 μm sterile filters and ultracentrifuged at 120,000 × g for 70 min. After careful removal of the supernatant, the exosome-containing pellet was resuspended in sterile PBS for downstream applications.

### 2.5 Characterization of the isolated serum-derived exosomes

The morphology, particle size and expression of surface markers of the isolated serum-derived exosomes were characterized by transmission electron microscope (TEM), NanoSight NS 300 system (NTA), and western blot. For TEM, the specific operation was as follows: add 20 μL of the prepared exosome suspension to a copper mesh for natural adsorption for 5–10 min. Then, use filter paper to absorb the excess liquid and let it dry. Subsequently, 20 μL of 2% phosphotungstic acid solution was added for staining for 3–5 min. The excess staining solution was then aspirated and dried under an incandescent lamp. Finally, the sample images were observed and captured using the JEM1400 transmission electron microscope from JEOL Company. For NTA, the sample was diluted with sterile PBS and filtered through a 0.22 μm filter to adjust the particle concentration to 10^8^–10^9^ particles /mL. According to the instructions, use a syringe to draw 1 mL of the sample and slowly inject it into the sample cell. After installing the laser module and inserting the thermometer probe, start the camera in the software, adjust the focal length and parameters, select the measurement mode and then run the test. Finally, the quantity and size of exosomes were detected using the NTA (NanoSight Technology, Malvern, United Kingdom), which is equipped with a green laser and a CMOS camera. For western blot, the exosomes were mixed with the sample loading buffer and then placed in a water bath at 100°C for 5–6 min. Then, the proteins were incubated with anti-CD9 antibody (1:1,000), anti-TSG101 antibody (1:1,000), anti-HSP70 antibody (1:1,000), and ani-GAPDH antibody (1:10,000) at 4°C overnight, followed by goat anti-rabbit IgG (H+L)-HRP (1:5,000) and goat anti-mouse IgG (H+L) -HRP (1:10,000) at 37°C for 1 h. After rinsing with 1 × PBST for 5 min for three times, protein bands were visualized using a chemiluminescence development.

### 2.6 Exosomal miRNA sequencing

Total RNA was extracted from the isolated serum-derived exosomes using mirVANA miRNA Isolation kit (Thermo Fisher Scientific, United States) following the manufacturer’s instructions. The concentrations and quality of the isolated RNA were determined using a NanoDrop 2000 (Thermo Fisher Scientific, MA, United States). RNA samples were commissioned to Xiamen Life Interconnection Technology Co., Ltd (Hangzhou, China) for miRNA sequencing (*n* = 3). After constructing the cDNA library using the TruSeq small RNA kit (Illumina, San Diego, United States), PE150 sequencing was performed on the Illumina NovaSeq 6000 platform. The original data was evaluated for quality by the fastqc software (version 0.11.2), which was used to remove the N base, filter out the low-quality sequence (q20), and cut off the connector. Subsequently, taking the GRCh38 genome (Ensembl release 102) as a reference, non-coding RNAs such as rRNA/tRNA were removed by bowtie alignment with the Rfam library. Finally, miRDeep2 (version 0.1.3) was used to identify the expression levels of miRNAs in all the samples.

### 2.7 Identification of DE-miRNAs and functional analyses

The expression levels were homogenized and differentially expressed using DESeq2 (version 3.21) in R 3.4.1. This project adopted the standards of false discovery rate (FDR) (the Benjamini and Hochberg method) < 0.05 and | log_2_ fold change (FC)| = 1 to screen differentially expressed miRNAs (DE-miRNAs). After that, the screened DE-miRNAs between the two groups were used to search their target genes using online tools, including predicted target genes, and experimentally verified target genes. Afterward, the predicted target genes, and experimentally verified target genes of DE-miRNAs were both submitted for functional analyses, containing gene ontology (GO), Kyoto Encyclopedia of Genes and Genomes (KEGG), Reactome Pathway Databas (Reactome), and Disease Ontology (DO). Finally, the DAVID 6.8 Bioinformatics Resources was used to visualized these pathways, and FDR < 0.05 was considered as the statistical significance.

### 2.8 RT-qPCR

The expression levels of the selected DE-miRNAs were determined using the stem-loop method. Briefly, the other three isolated total RNA were reverse transcribed into cDNA using the PrimeScript*™* II first-strand cDNA synthesis kit (Taraka, Japan) in line with the manufacturer’s instructions. The cDNA synthesis reaction was performed in a 10 μL system containing 1 μL RT-Primer, 1 μL dNTP mixture, 500 ng RNA, and RNase-free H_2_O. The mixture was incubated at 65°C for 5 min, followed by the addition of 4 μL 5 × PrimeScript II Buffer, 0.5 μL RNase Inhibitor, 1 μL PrimeScript II RTase, and 4.5 μL RNase-free H_2_O. The obtained mixture was incubated at 42° for 60 min and then at 95° for 5 min in sequence. Finally, qPCR amplification was carried out using Power SYBR Green PCR Master Mix (Thermo Fisher Scientific) following the supplier’s protocols. The sequences of all primers are shown in Table 2, and U6 was used as the internal reference gene. The relative expression levels of hsa-miR-100-5p, hsa-miR-23b-3p, hsa-miR-373-3p, and hsa-miR-27b-3p were calculated by the 2^–Δ^ ^Δ^ ^Ct^ method.

**TABLE 2:** Details of PCR primers utilized in this investigation.

Primer	Sequences (5′–3′)
hsa-miR-100-5p	RT: GTCGTATCCAGTGCAGGGTCCGAGGTATTCGCACTGGATACGAC TTCAGT
	F: GCGCCGCTACAGTACTGTGATA
hsa-miR-23b-3p	RT: GTCGTATCCAGTGCAGGGTCCGAGGTATTCGCACTGGATACGAC GTGGTA
	F: GCGATCACATTGCCAGGGAT
hsa-miR-373-3p	RT: GTCGTATCCAGTGCAGGGTCCGAGGTATTCGCACTGGATACGAC GCAGAA
	F: GCGCGAAGTGCTTCGATTTTG
hsa-miR-27b-3p	RT: GTCGTATCCAGTGCAGGGTCCGAGGTATTCGCACTGGATACGA CGCAGAA
	F: GCGCGTTCACAGTGGCTAAG
Downstream universal primer	GTGCAGGGTCCGAGGT
U6	RT: GTCGTATCCAGTGCAGGGTCCGAGGTATTCGCACTGGATACGAC AAAATATG
	F: CTCGCTTCGGCAGCACA
	R: AACGCTTCACGAATTTGCGT

### 2.9 Statistical analysis

Data were described as mean ± standard deviation (SD) with triple repetition, and statistical analysis was conducted using GraphPad Prism 5 (Graphpad Software, San Diego, CA). For comparison for two groups, the student’s *t*-test was utilized to evaluate the differences. Statistical significance was defined as *P* < 0.05.

## 3 Results

### 3.1 Exosomes successfully isolated from serum of ARHL patients and healthy controls

**FIGURE 1:**
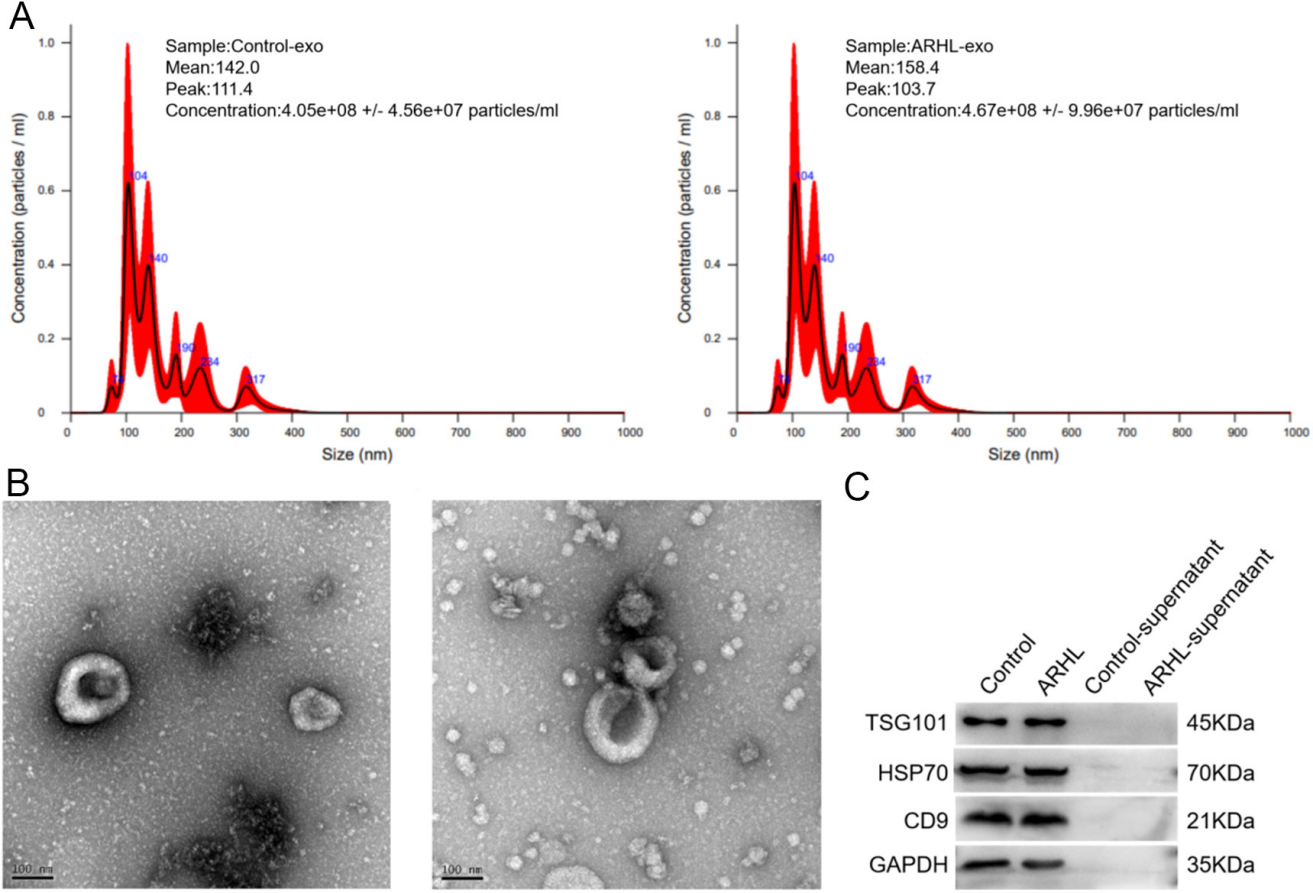
Identification of exosomes isolated from serum of control individuals and ARHL patients. **(A)** Particle distribution and an average diameter of exosomes measured by NTA. **(B)** TEM revealed spherical or disc-shaped of exosomes with a double-membrane structure. **(C)** Western blot showed TSG101, HSP70 and CD9 were all expressed. Control, exosomes isolated from control individuals; ARHL, exosomes isolated from ARHL patients; Control-supernatant, supernatant from control individuals; ARHL-supernatant, supernatant from ARHL patients.

In order to explore the exosomal mechanisms of ARHL, exosomes were isolated from serum samples of ARHL patients and healthy controls, and then were characterized by TEM, NTA and western blot. NTA results revealed that the major peaks in particle size of the control serum-derived exosomes and ARHL serum-derived exosomes were respectively about 111.4 and 103.7 nm; and the mean peaks of the control serum-derived exosomes and ARHL serum-derived exosomes were approximately 142 and 158.4 nm (Figure 1A), which was consistent with the previously reported exosomes size distributions (Hade et al., 2021; Isaac et al., 2021). Then, TEM showed that exosomes isolated from control and ARHL serum were both spherical or disc-shaped with a diameter of about 150 nm, and had a bilayer membrane structure (Figure 1B). Furthermore, western blot analysis confirmed the presence of exosomal marker proteins TSG101, HSP70, and CD9 in the isolated substances, while these markers were absent in the supernatant (Figure 1C). These results indicated that exosomes were successfully isolated from the serum of the ARHL patients and healthy controls with this ultracentrifugation method.

### 3.2 Quality control of sequence reads and annotation of miRNAs

**FIGURE 2:**
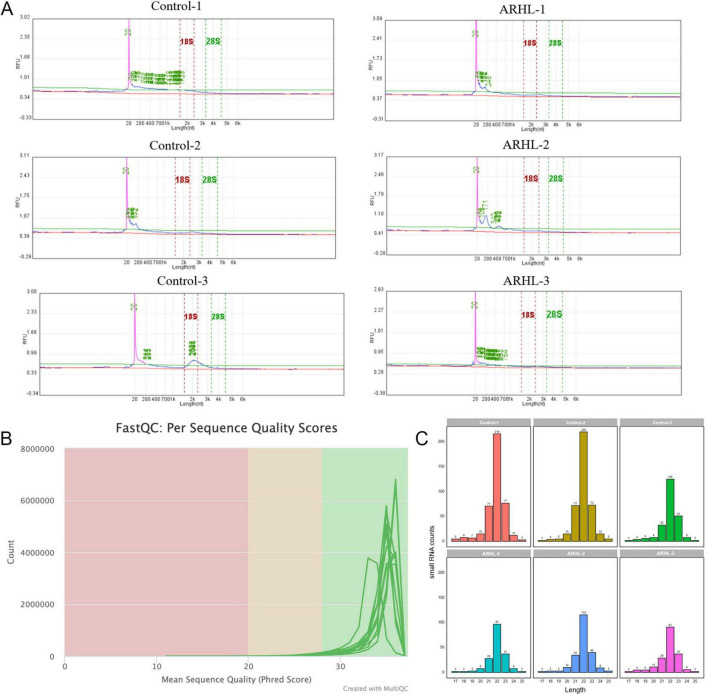
Quality control of sequence reads and identification of microRNAs (miRNAs). **(A)** The results of exosomal RNA Qsep100. The horizontal axis represents the length of RNA, and the vertical axis represents the fluorescence intensity of the RNA sample. **(B)** Distribution of sequencing quality. **(C)** Statistics of miRNA length expressed.

Exosomal RNA sequencing requires a minimum input of over 1 ng RNA for library construction, with a total library quantity exceeding 10 ng being adequate for subsequent sequencing. The RNA concentrations isolated from each sample ranged from 0.19 to 0.28 ng/μL, as well as the RNA amount were 5.13–7.56 ng (Table 3). The library concentrations of each sample were 4.99–9.7 ng/μL, as well as the library amount ranged from 69.86 to 135.8 ng (Table 3). These results indicated that the quantities of extracted RNA satisfied these criteria for both library preparation and sequencing. Additionally, Qsep100 analysis verified the integrity of the isolated exosomal RNA and ruled out potential cellular contamination (Figure 2A).

**TABLE 3:** The RNA concentration of exosomes constructed by the library, and the quality of the constructed library.

Sample	RNA concentration (ng/μ L)	RNA amount (ng)	Library concentration (ng/μ L)	Library amount (ng)
Control-1	0.24	6.48	5.7	79.8
Control-2	0.26	7.02	5.7	79.8
Control-3	0.2	5.40	4.99	69.86
ARHL-1	0.28	7.56	7.5	105
ARHL-2	0.23	6.21	9.7	135.8
ARHL-3	0.19	5.13	6.2	86.8

Quality control metrics confirmed that each sample generated more than 10 million clean reads (Table 4). After aligning with the Rfam database using Bowtie, we identified and filtered out rRNA and other non-coding RNAs (ncRNAs), with each sample containing fewer than 500,000 ncRNA reads (Table 5). Subsequent alignment to the reference genome showed that all samples had mapping rates above 25% (Table 6), confirming the high quality of the sequencing data, which is suitable for further analysis.

**TABLE 4:** Quality assessment of sample sequencing output data.

File	Reads_raw	Q20_raw	Q30_raw	Raw_GC%	Reads_clean	Q20_clean	Q30_clean	Clean_GC%
Control-1_R1	10,193,022	94.41%	88.79%	57%	10,103,573	99.10%	97.07%	48%
Control-1_R2	10,193,022	97.01%	93.13%	54%	10,103,573	99.28%	97.39%	48%
Control-2_R1	10,330,606	94.14%	87.77%	57%	10,246,060	98.39%	94.82%	48%
Control-2_R2	10,330,606	95.16%	89.68%	54%	10,246,060	98.87%	95.92%	48%
Control-3_R1	11,486,268	95.60%	90.73%	57%	11,379,234	99.04%	96.98%	48%
Control-3_R2	11,486,268	96.42%	92.20%	56%	11,379,234	99.16%	97.04%	48%
ARHL-1_R1	10,638,501	89.52%	82.45%	59%	10,549,930	98.45%	94.92%	48%
ARHL-1_R2	10,638,501	95.47%	90.26%	57%	10,549,930	98.98%	96.30%	48%
ARHL-2_R1	12,157,984	93.70%	87.05%	59%	12,046,412	98.27%	94.60%	48%
ARHL-2_R2	12,157,984	94.81%	89.04%	56%	12,046,412	98.67%	95.60%	48%
ARHL-3_R1	10,379,344	95.03%	89.21%	59%	10,289,895	98.56%	95.20%	48%
ARHL-3_R2	10,379,344	95.45%	90.18%	56%	10,289,895	98.95%	96.01%	48%

GC represents the GC content of the sequence. Raw refers to the offline data, and clean represents the sequence after quality control filtering.

**TABLE 5:** Statistics of Rfam database comparison.

No	Sample	Cis-reg	lncRNA	Other sRNA	Others	rRNA	snoRNA	Total
1	Control-1	2,120	126	2,426	379,097	81,029	71	464,869
2	Control-2	3,742	116	3,922	345,956	76,574	230	430,540
3	Control-3	2,567	348	2,704	244,334	83,931	220	334,104
4	ARHL-1	3,647	179	3,182	277,009	66,654	294	350,965
5	ARHL-2	4,123	307	3,256	343,222	62,073	241	413,222
6	ARHL-3	3,230	195	3,467	337,760	94,695	417	439,764

**TABLE 6:** Statistics of read alignment rates after Rfam library filtration.

File	Qualified_reads	Mapped_reads	Map_rate
Control-1	3,830,917	955,673	25%
Control-2	4,708,717	1,233,292	26%
Control-3	4,178,215	1,160,374	28%
ARHL-1	6,562,389	2,545,064	39%
ARHL-2	7,742,422	4,602,960	59%
ARHL-3	4,755,286	1,263,598	27%

In addition, the overall base quality of the sequencing data met the Q30 standard (Figure 2B). Read length distribution analysis indicated that most reads were 20–24 nt in length, with a prominent peak at 22 nt (Figure 2C).

### 3.3 16 DE-miRNAs identified between the exosomes from controls and ALHR samples

**FIGURE 3:**
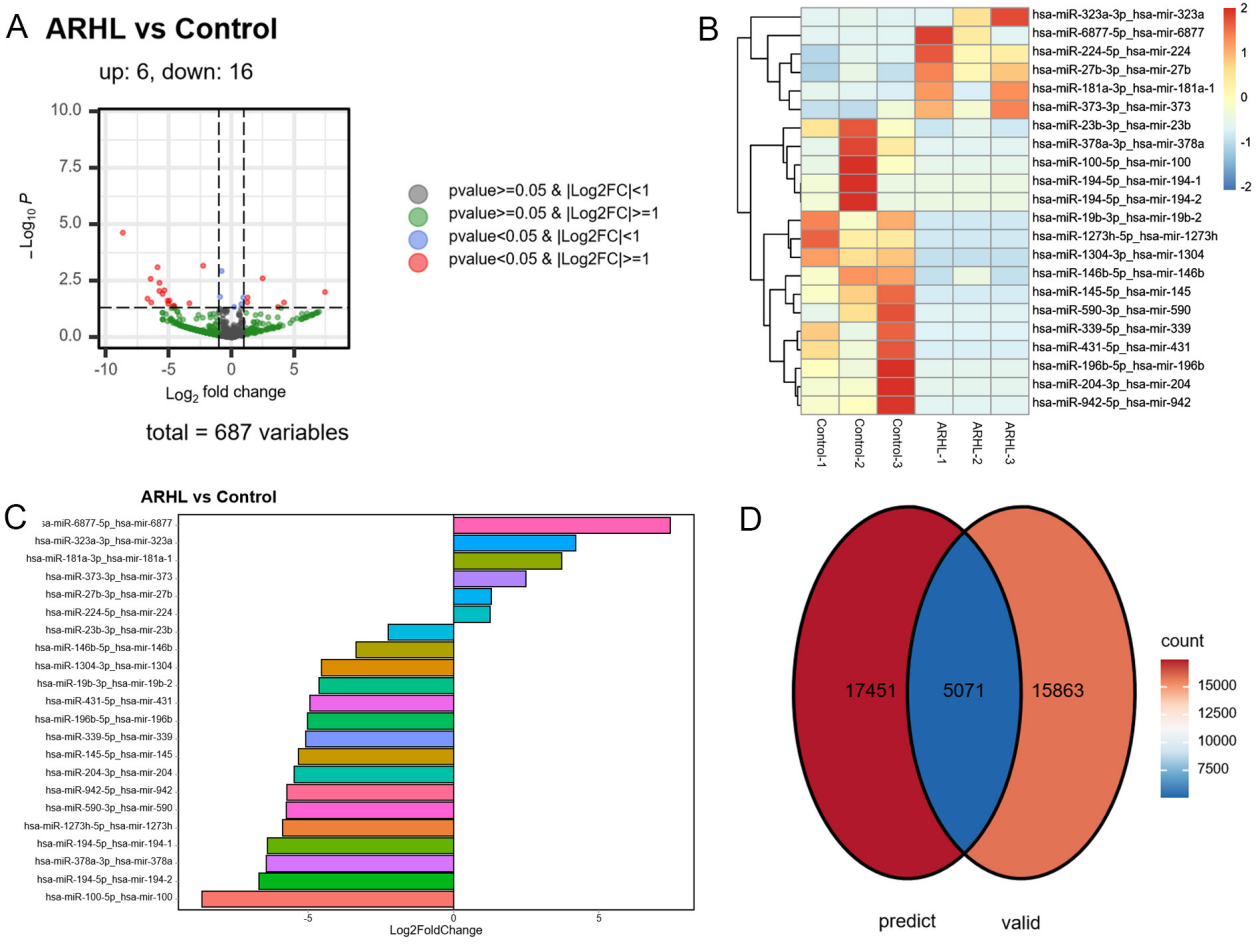
Screening of differentially expressed miRNAs (DE-miRNAs). **(A)** The expression volcano diagram of DE-miRNAs. **(B)** The expression heatmap of DE-miRNAs. **(C)** The expression bar chart of DE-miRNAs (To the right is an upward adjustment, and to the left is a downward adjustment). **(D)** Venn diagram of the intersection of the predicted target genes and the experimentally verified target genes.

According to the thresholds of log_2_FC = 1 and FDR < 0.05, a total of 22 DE-miRNAs were identified in the ALHR and elderly control samples, among which 6 were upregulated (hsa-miR-323a-3p, hsa-miR-6877-5p, hsa-miR-224-5p, hsa-miR-27b-3p, hsa-miR-181a-3p, and hsa-miR-373-3p) and 16 were downregulated (hsa-miR-23b-3p, hsa-miR-378a-3p, hsa-miR-100-5p, hsa-miR-194-5p (precursors are hsa-mir-194-1 and hsa-mir-194-2, respectively), hsa-miR-19b-3p, hsa-miR-1273h-5p, hsa-miR-1304-3p, hsa-mir-146b-5p, hsa-miR-145-5p, hsa-miR-590-3p, hsa-miR-339-5p, hsa-miR-431-5p, hsa-miR-196b-5p, hsa-miR-204-3p, hsa-miR-942-5p) (Figure 3A). The heatmap of the identified DE-miRNAs revealed that the identified DE-miRNAs could clearly distinguish the ALHR samples from the controls (Figure 3B). The bar chart provided a more intuitive visualization of the differential expression changes for each DE-miRNA (Figure 3C). Online searches for the target genes of DE mature miRNAs revealed that these DE-miRNAs collectively identified 17,451 predicted target genes and 15,863 experimentally validated target genes (Figure 3D).

### 3.4 Enrichment analyses of the target genes

The predicted and experimentally verified target genes of the identified DE-miRNAs were respectively subjected to enrichment analyses, including GO, KEGG, Reactome and DO. In addition to enriching analyses of cytological functions and signaling pathway functions, we performed DO enrichment analysis on the target genes. This strategy sought to explore the potential involvement of DE-miRNAs in diverse diseases via the regulation of their target genes, and to examine whether common molecular mechanisms or pathways exist across these diseases.

**FIGURE 4:**
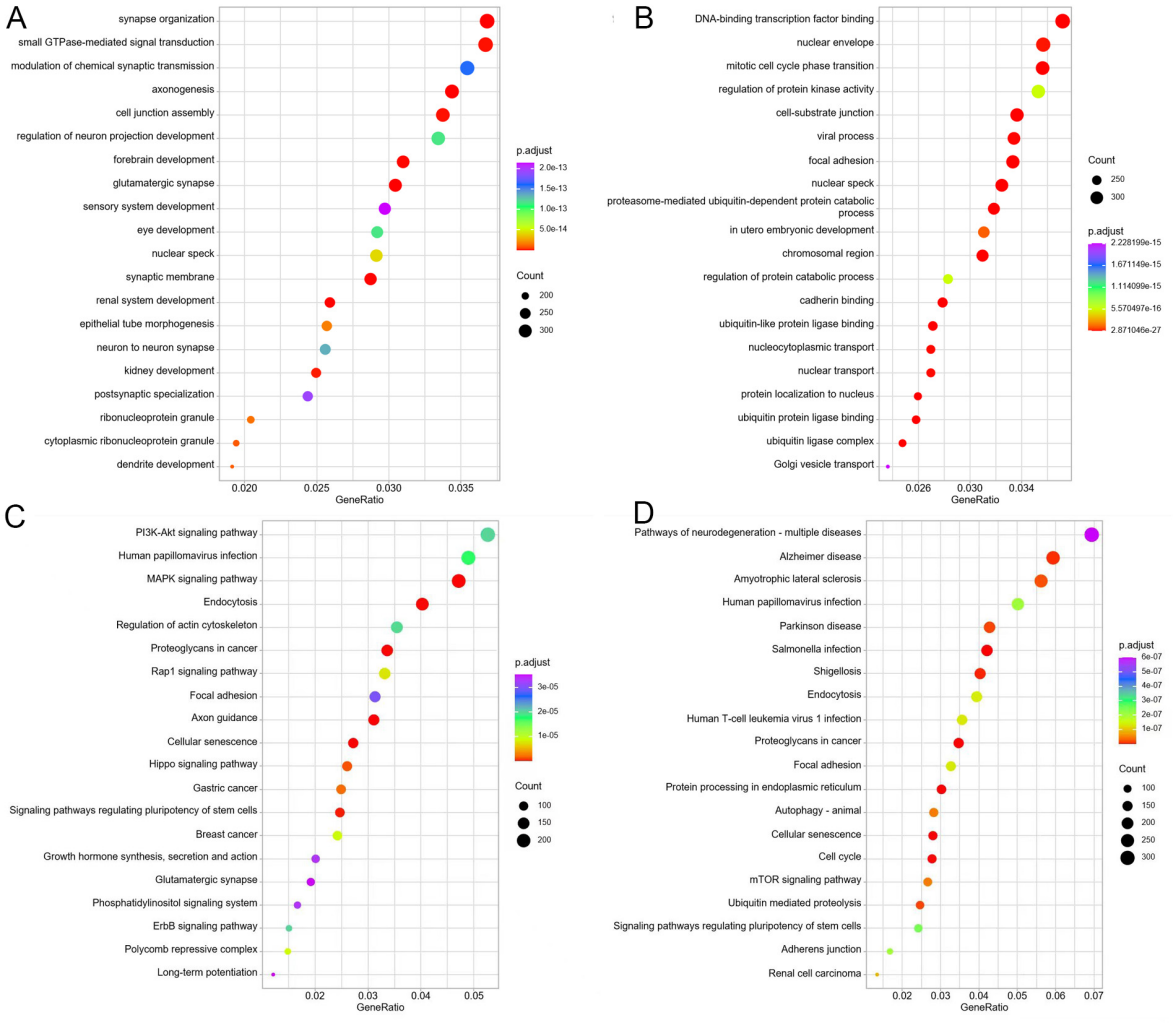
GO and KEGG functional analyses of the screened target genes of DE-miRNAs. **(A)** Top 20 significantly GO terms enriched by the predicted target genes of DE-miRNAs. **(B)** Top 20 significantly GO terms enriched by the verified target genes of DE-miRNAs. **(C)** Top 20 significantly KEGG terms enriched by the predicted target genes of DE-miRNAs. **(D)** Top 20 significantly KEGG terms enriched by the verified target genes of DE-miRNAs.

GO enrichment analysis of the predicted target genes showed significant enrichment in several critical biological processes and cellular functions, including “synapse organization,” “small GTPase-mediated signal transduction,” “modulation of chemical synaptic transmission,” “axonogenesis,” “cell junction assembly,” “regulation of neuron projection development,” “forebrain,” “glutamatergic synapse,” “sensory system development,” “neuron to neuron synapse,” and “dendrite development” (Figure 4A). Furthermore, the verified target genes of the identified DE-miRNAs were significantly enriched in “DNA-binding transcription factor binding,” “nuclear envelope,” “mitotic cell cycle phase transition,” “regulation of protein kinase activity,” “cell-substrate junction,” “viral process,” “proteasome-mediated ubiquitin-dependent protein catabolic,” “chromosomal region,” “regulation of protein catabolic process,” and “ubiquitin-like protein ligase binding” (Figure 4B).

The KEGG functional enrichment analysis of the target genes showed that the predicted target genes were significantly enriched in several pathways, such as “PI3K-Akt signaling pathway,” “human papillomavirus infection,” “MAPK signaling” “pathway,” “endocytosis,” “regulation of actin cytoskeleton,” “Rap1 signaling pathway,” “axon guidance,” “cellular senescence,” “Hippo signaling pathway,” “signaling pathways regulating pluripotency of stem cells,” “ErbB signaling pathway,” and “phosphatidylinositol signaling pathway” (Figure 4C). In addition, the verified target genes exhibited significant enrichment in pathways related to “pathways of neurodegeneration—multiple disease,” “endocytosis,” “protein processing in endoplasmic reticulum,” “autophagy-animal,” “cellular senescence,” “mTOR signaling pathway,” “ubiquitin mediated proteolysis,” “signaling pathway regulating pluripotency of stem cells,” “cell cycle,” and “adherens junction” (Figure 4D).

**FIGURE 5:**
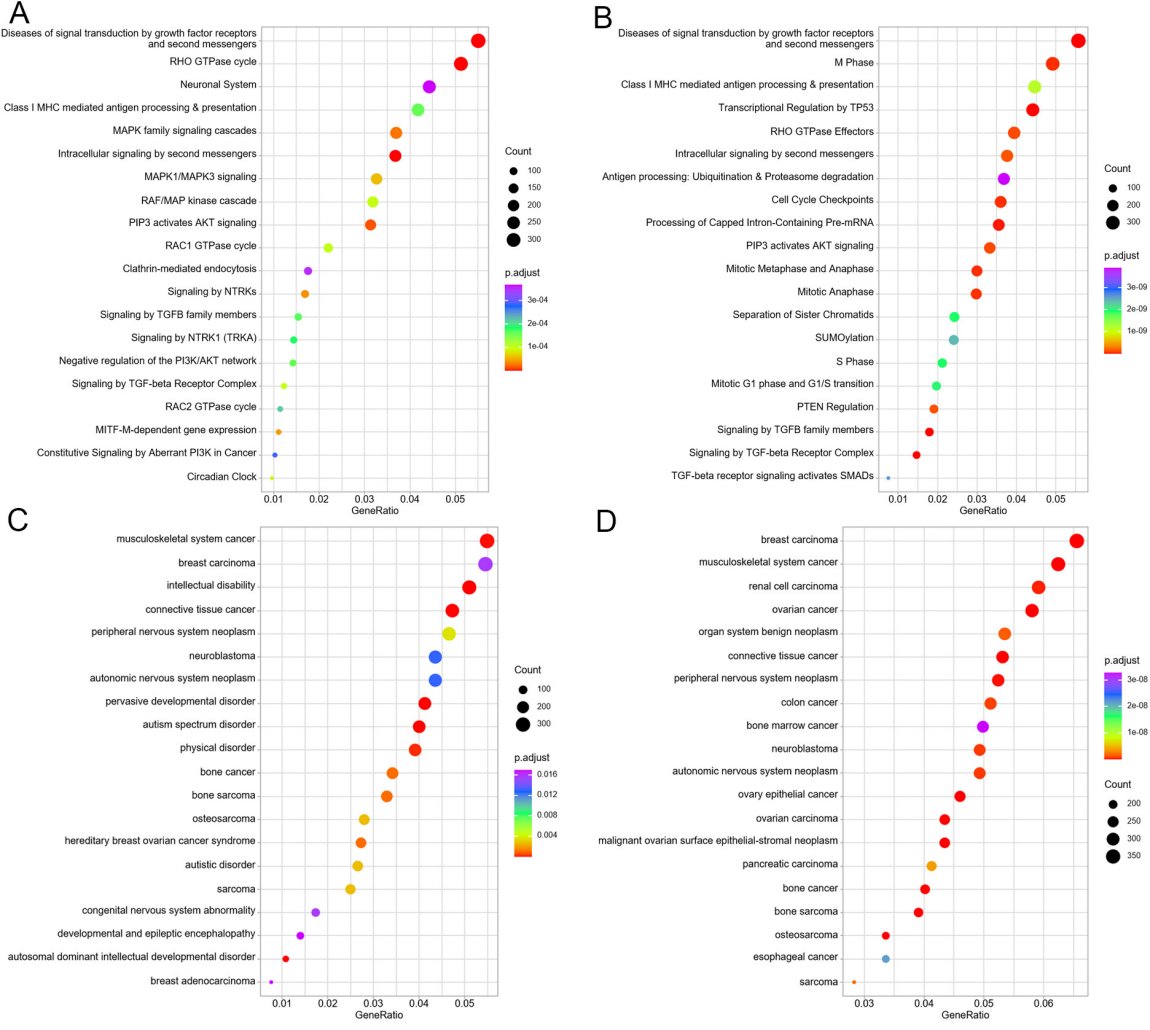
Reactome and disease ontology (DO) functional analyses of the screened target genes of DE-miRNAs. **(A)** Top 20 significantly Reactome terms enriched by the predicted target genes of DE-miRNAs. **(B)** Top 20 significantly Reactome terms enriched by the verified target genes of DE-miRNAs. **(C)** Top 20 significantly DO terms enriched by the predicted target genes of DE-miRNAs. **(D)** Top 20 significantly DO terms enriched by the verified target genes of DE-miRNAs.

Reactome pathway enrichment analysis demonstrated significant enrichment of the predicted target genes in key biomolecular pathways including “diseases of signal transduction by growth factor receptor and second messenger,” “RHO GTPase cycle,” “MAPK family signaling cascade,” “neuronal system,” “MAPK1/MAPK3 signaling,” “RAF/MAP kinase cascade,” “PIP3 activates AKT signaling,” “signaling by NTRKs,” “negative regulation of the PI3K/AKT network,” “signaling by TGF-β receptor complex,” and “MITF-M-dependent gene expression”(Figure 5A). The verified target genes were significantly enriched in “diseases of signal transduction by growth factor receptor and second messengers,” “M Phase,” “Class I MHC mediated antigen processing and presentation,” “transcriptional regulation by TP53,” “RHO GTPase effectors,” “antigen processing: ubiquitination and proteasome degradation,” “signaling by TGFB family members,” “intracellular signaling by second messengers,” “signaling by TGFB family members,” “TGF-beta receptor signaling activates SMADs,” and “SUMOylation” (Figure 5B).

In addition, the DO functional enrichment analysis of the target genes displayed that the predicted target genes were significantly associated with “intellectual disability,” “peripheral nervous system neoplasm,” “neuroblastoma,” “autonomic nervous system neoplasm,” “pervasive developmental disorder,” “autosomal dominant intellectual developmental disorder,” “physical disorder,” “autistic disorder,” “congenital nervous system abnormality,” and “developmental and epileptic encephalopathy” (Figure 5C). The verified target genes demonstrated significant enrichment in diseases or pathological states such as “organ system benign neoplasm,” “peripheral nervous system neoplasm,” “neuroblastoma,” “autonomic nervous system neoplasm,” and “malignant ovarian surface epithelial-stromal neoplasm” (Figure 5D).

### 3.5 Construction of two miRNA-target regulatory networks

**FIGURE 6:**
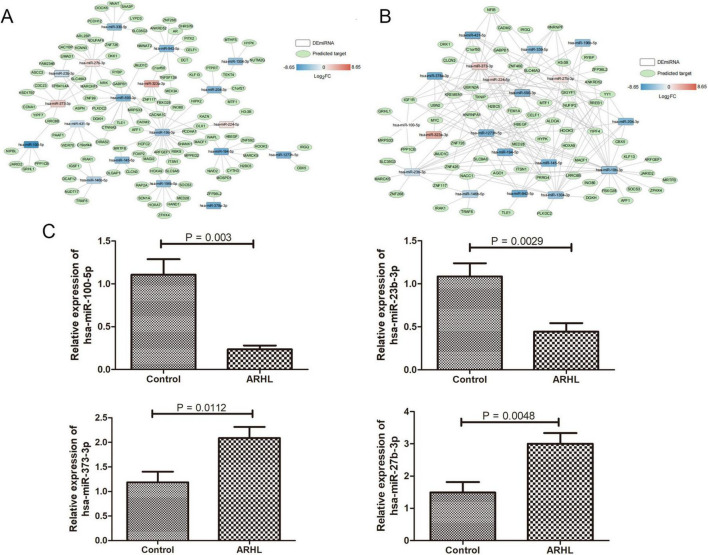
The co-expression networks of DE-miRNAs and screened target genes, as well as experimental verification. **(A)** The co-expression networks of DE-miRNAs and predicted target genes. The rectangle and oval represent miRNAs and mRNAs, respectively. **(B)** The co-expression networks of DE-miRNAs and verified target genes. The rectangle and oval represent miRNAs and mRNAs, respectively. The color indicates the significant difference of each transcript. **(C)** Expression levels of exosomal hsa-miR-100-5p, hsa-miR-23b-3p, hsa-miR-373-3p, and hsa-miR-27b-3p in control and ARHL-derived exosomes. *N* = 3. Data were expressed mean ± standard deviation (SD).

Using stringent criteria, we systematically constructed two miRNA-target regulatory networks. First, we ranked the prediction scores from each database in descending order, filtered for high-confidence predictions, and built a DE-miRNA-predicted target gene regulatory network comprising 141 pairs of significant regulatory interactions (Figure 6A). In this DE-miRNA-predicted target gene regulatory network, hsa-miR-19b-3p and hsa-miR-500-3p were the hub nodes. Second, by strictly selecting interactions supported by experimental validation, support_type, and pubmed_id literature evidence, we constructed a highly reliable DE-miRNA-verified target gene regulatory network containing 189 interaction pairs (Figure 6B). In this DE-miRNA-verified target gene regulatory network, hsa-miR-1273h-5p, hsa-miR-500-3p, hsa-miR-224-5p, and hsa-miR-373-3p were the hub nodes.

3.6 Down-regulated hsa-miR-100-5p, hsa-miR-23b-3p, and up-regulated hsa-miR-373-3p, hsa-miR-27b-3p in ALHR exosomes using RT-qPCR

Four DE-miRNAs (hsa-miR-100-5p, hsa-miR-23b-3p, hsa-miR-373-3p, and hsa-miR-27b-3p) were selected for RT-qPCR analysis. It was found that compared with the serum-derived exosomes from the controls, the expression levels of hsa-miR-100-5p (*P* = 0.003) and hsa-miR-23b-3p (*P* = 0.0029) were significantly reduced, while the expression levels of hsa-miR-373-3p (*P* = 0.0112) and hsa-miR-27b-3p (*P* = 0.0048) were evidently elevated in the serum-derived exosomes from the ALHR patients (*P* < 0.05) (Figure 6C). The differential expression patterns verified by RT-qPCR were consistent with the sequencing result, which implied a high relatively reliability of the sequencing results.

## 4 Discussion

The pathogenesis of ARHL is complex and highly heterogeneous. Current clinical diagnosis and therapeutic evaluation primarily rely on a comprehensive assessment approach combining clinical symptoms with audiometric findings. There remains an urgent clinical need to develop functional and molecular biomarkers for detecting reversible preclinical pathological changes, as well as to establish effective intervention strategies to address these challenges in clinical management. ARHL is influenced by a series of external and internal factors, including long-term exposure to external noise (Otte et al., 1978), ototoxic medication use (Gratton et al., 1996), cochlear vascular pathologies and hemodynamic alterations (Gratton et al., 1997), neurotransmitter activity modifications (Sha et al., 2008), dietary habits (Gratton et al., 1997), oxidative stress (Ohlemiller et al., 2010), and mitochondrial gene deletions (Engle et al., 2013), methylation epigenetic modifications (Schuknecht and Gacek, 1993), etc. Despite these identified risk factors, the precise molecular mechanisms underlying ARHL pathogenesis remain poorly understood.

As an important regulatory factor for the occurrence and development of deafness, the role of miR-204 and its subtypes in the auditory system has been confirmed by multiple studies. Studies have shown that miR-204-5p is significantly upregulated in the cochlear tissues of aged mice, exerting a protective effect by inhibiting H_2_O_2_-induced apoptosis of HEI-OC1 cells and promoting cell proliferation (Hu et al., 2024). In the acute hearing loss model, miR-204-5p can interact with the transcription factor Sp1 to alleviate hypoxia or LPS-induced damage to HEI-OC1 cells (Xie et al., 2021). Furthermore, miR-204-5p modulates spiral ganglion neuron viability in a concentration-dependent manner through targeted regulation of TMPRSS3 expression, with its inhibition promoting neuronal functional recovery and ameliorating sensorineural hearing loss (Jiang et al., 2020; Li et al., 2014). Intriguingly, both miR-204-5p and miR-204-3p show significant downregulation in hippocampal tissues following noise-induced hearing loss (Ha et al., 2021). Our study reveals for the first time a marked reduction of exosomal miR-204-3p in serum samples from presbycusis patients, providing novel clues into the molecular mechanisms of ARHL, although the precise underlying mechanisms require further investigation.

Accumulating evidence highlights the crucial role of miR-431 in mammalian inner ear development and auditory function maintenance. During cochlear development, miR-431 exhibits high expression levels in spiral ganglion neurons of neonatal mice, with progressive downregulation throughout maturation. Notably, miR-431 overexpression leads to significant reductions in spiral ganglion neuron density and subsequent hearing impairment (Fan et al., 2016). In noise-induced hearing loss models, animals with miR-431 overexpression demonstrate more severe auditory dysfunction accompanied by greater loss of cochlear inner hair cell ribbon synapses compared to controls, suggesting miR-431 may enhance cochlear vulnerability to acoustic trauma (Fujita et al., 2023). Multivariate analysis further confirmed that miR-431 was independently associated with hearing loss at 6 months (Huang et al., 2025). Our current study reveals significantly decreased miR-431 expression in serum exosomes from elderly subjects, implying its potential involvement in the pathogenesis of ARHL.

In addition, our RT-qPCR validation confirmed that hsa-miR-100-5p and hsa-miR-23b-3p were significantly downregulated, while hsa-miR-373-3p and hsa-miR-27b-3p were upregulated in serum exosomes of ARHL patients, consistent with sequencing data. Hsa-miR-100-5p, down-regulated in ARHL-associated exosomes, is a known modulator of mTOR signaling (Liu et al., 2024a), a pathway previously implicated in ARHL via its regulation of cochlear hair cell autophagy and senescence (Ege et al., 2024). A previous study of Wang et al. (Wang and Liu, 2025) demonstrated that miRNA-634 was highly expressed in exosomes extracted from the peripheral blood samples of ARHL patients, and could promote apoptosis of HEI-OC1 cells by regulating the AKT/mTOR signaling pathways, thereby influencing the progression of ARHL. It has been discovered that the hsa-miR-23b-3p can prevent neuronal cell apoptosis and the production of reactive oxygen species (Zhan et al., 2016). Itokazu et al. demonstrated that the expression of miR-23b-3p in exosomes decreased with the increase of donor age, as well as *TGFBR3* was identified as the key target of miR-23b-3p, and its inhibition led to the upregulation of multiple genes related to the stemness characteristics of mesenchymal stem cells (MSCs), suggesting exosomes delivered miR-23b-3p could serve as a biomarker for predicting the therapeutic effect of MSC treatment (Itokazu et al., 2025). The roles of hsa-miR-373-3p have been widely reported in many cancers, including cervical cancer, pancreatic carcinoma, and choriocarcinoma (Hu et al., 2018; Lu et al., 2021; Yu et al., 2021). It has been reported that high level of miR-373-3p could enhance lactate production by targeting *MFN2*, thus promoting aerobic glycolysis and proliferation of colon cancer cells (Wang et al., 2024a). In addition, PPAR signaling pathway is crucial for ARHL, and miR-27b-3p is predicted to regulate PPARG, which indicated miR-27b-3p and PPARG may be potential strategies for ARHL treatment (Zhang et al., 2019). Taken together, it can be inferred that hsa-miR-100-5p, hsa-miR-23b-3p, hsa-miR-373-3p, and hsa-miR-27b-3p may be potential crucial regulators of ARHL, due to related pathways critical for auditory homeostasis, neuroprotection, and senescence. Research has found that ubiquitinated proteins accumulate in the cochlea of aged mice, and endoplasmic reticulum stress impairs the function of clearing abnormal proteins through the ubiquitin-proteasome system (Wang et al., 2015). The deubiquitinating enzyme UCHL1 is down-regulated in H2O2-induced HEI-OC1, while its overexpression can enhance cell viability, alleviate oxidative damage, apoptosis and senescence (Li et al., 2023). Fbx2 is a ubiquitin ligase F-box protein that is highly expressed in Corti organs. Mice with targeted deletion of the Fbxo2 gene began to show age-related hearing loss at 2 months (Nelson et al., 2007). Our current findings further support the pivotal role of ubiquitination in ARHL. Bioinformatics analysis demonstrated that predicted target genes of DE-miRNAs were significantly enriched in key ubiquitin-related processes, including “proteasome-mediated ubiquitin-dependent protein catabolism” and “ubiquitin-like protein ligase binding” (GO terms), as well as the “ubiquitin mediated proteolysis” pathway (KEGG). Additionally, validated target genes showed enrichment in the Reactome pathway of “antigen processing: ubiquitination and proteasome degradation.” These consistent findings across multiple analytical approaches strongly implicate ubiquitination dysfunction as a critical mechanism underlying ARHL.

Cellular senescence has been implicated in the pathogenesis of various age-related degenerative disorders, including ARHL (Chen et al., 2025). In ARHL, cellular senescence manifests not only through morphological alterations such as progressive loss of afferent and efferent synapses, but also via functional declines characterized by reduced K^+^ currents and diminished nonlinear capacitance (Jeng et al., 2020). Oxidative stress-induced premature senescence of auditory cells represents a key contributor to ARHL development (Rivas-Chacón et al., 2021). Experimental studies using H_2_O_2_-induced senescence models in HEI-OC1 cells demonstrate that mitigation of mitochondrial oxidative stress significantly attenuates senescence-associated phenotypes and delays auditory cell aging (Rivas-Chacón et al., 2023). Our current findings further corroborate the pivotal role of cellular senescence signaling pathways in the occurrence of ARHL.

The changes in autophagy are closely related to the occurrence of presbycusis (Fujimoto et al., 2017). Oxidative stress impairs autophagic flux in auditory hair cells, leading to premature senescence characteristics including nuclear deformation and reduced proliferation capacity (Tsuchihashi et al., 2015). Neurotransmitters significantly inhibit D-galactose-induced hair cell loss by promoting autophagic influx (Wang et al., 2025). The dual leucine zipper kinase pathway may contribute to cochlear hair cell senescence and ARHL progression through aberrant activation of autophagy in hair cells (Ding et al., 2025). Overexpression of RONIN increased autophagy levels and weakened hair cell senescence induced by D-galactose (Wei et al., 2025). Our previous animal studies have substantiated the critical involvement of autophagy in ARHL development (Zhang et al., 2024). The current study identified significant enrichment of validated target genes from DE-miRNAs in autophagy-related KEGG pathways. Currently, studies related to human samples and the autophagy pathway are still relatively scarce.

Previous studies have found that PIN1 mediates the senescence of cochlear hair cells through the PI3K/Akt/mTOR signaling pathway (Zhang et al., 2021). Notably, 17β-estradiol exerts protective effects against ARHL by promoting angiogenesis via PI3K/AKT activation (Feng et al., 2021). Furthermore, rapamycin ameliorates presbycusis by enhancing autophagy in spiral ganglion neurons through mTOR inhibition (Liu et al., 2022). Based on our current research, it is suggested that PI3K/AKT and mTOR signals also play significant roles in ARHL development.

Our GO analysis identified “regulation of neuron projection development,” “sensory system development,” and “dendrite development” as top-enriched terms for DE-miRNA target genes, pathways critical for maintaining central auditory pathway integrity. ARHL is not merely a peripheral cochlear disorder; central auditory processing deficits (e.g., impaired speech recognition, delayed auditory reaction time) are increasingly recognized as key clinical features, driven by structural and functional disruptions in the auditory cortex, cochlear nucleus, and hippocampus (Jayakody et al., 2018; Sardone et al., 2019). In addition, the DO analysis also revealed significant enrichment of both predicted and validated target genes in nervous system-related categories, strongly suggesting an association between ARHL and neurological dysfunction. Existing research indicates that the pathological mechanism of presbycusis involves multi-level neurological functional abnormalities: in the peripheral auditory system, the loss of synapses between inner hair cells and spiral ganglion neurons is an early feature of cochlear aging and preceded the loss of neurons and hair cells (Wang et al., 2024b). In the auditory central system, the expressions of complement components (C1q, C3) and pro-inflammatory cytokines (TNF-α, IL-1β) in the cochlear nucleus were significantly upregulated, indicating that neuroinflammatory responses and complement system activation may be involved in the disease process (Cassinotti et al., 2022). At the neuroelectrophysiological level, the discharge frequency of central neurons and the intensity of excitatory postsynaptic currents show an age-dependent decrease under continuous acoustic stimulation (Wang et al., 2019). In higher auditory centers, ARHL patients exhibit not only an inverse correlation between hippocampal gray matter volume and hearing thresholds (Shim et al., 2023), but also altered spontaneous neural activity characterized by decreased activity in the superior temporal gyrus, parahippocampal gyrus, precuneus, and inferior parietal lobule, coupled with increased activity in the middle frontal gyrus, cuneus, and postcentral gyrus (Chen et al., 2018). Collectively, these data indicated that DE-miRNA-mediated disruptions in nervous system development neurological functions may contribute to both peripheral and central ARHL pathology. The clinical management of ARHL currently lacks non-invasive early diagnostic tools, relying primarily on pure-tone audiometry that only detects hearing loss post-irreversible cochlear damage, but our identification of serum exosomal miRNAs (hsa-miR-100-5p, hsa-miR-23b-3p, hsa-miR-373-3p, hsa-miR-27b-3p) offers a promising liquid biopsy solution with unique advantages for ARHL. Exosomal miRNAs are inherently stable in serum [protected by the lipid bilayer from RNase degradation (Katayama et al., 2025)], robust to pre-analytical variability (e.g., sample storage, processing time), a critical clinical biomarker requirement, and serum exosomes are easily obtainable via peripheral venous blood (unlike inaccessible cochlear tissue biopsies in living patients), enabling repeated sampling for longitudinal monitoring. This is particularly valuable for gradually progressive ARHL, as serial measurement of the four key miRNAs could detect early pathological changes (e.g., hsa-miR-100-5p downregulation preceding threshold shifts) before clinical symptoms emerge. Additionally, the four miRNAs show strong discriminatory power: our RT-qPCR data revealed significant expression differences between ARHL and control groups (all *P* < 0.01), and their combined signature may improve diagnostic accuracy. Finally, exosomal miRNAs may enable personalized therapeutic response monitoring. For example, if future studies validate mTOR inhibitors (e.g., rapamycin) as ARHL treatments (Li et al., 2025; Liu et al., 2022), increased hsa-miR-100-5p expression may act as a pharmacodynamic marker of efficacy, indicating restored mTOR regulation and autophagy. Therefore, these features position serum exosomal miRNAs as transformative tools for ARHL’s early diagnosis, prognosis, and therapeutic monitoring, addressing an unmet clinical need.

However, this study has several limitations that need to be acknowledged. First, the sample size for the core miRNA sequencing experiment was relatively small (*n* = 3 per group), which is a common constraint in initial high-throughput discovery studies but may increase the risk of false positive or false negative results. The small sample size limits the statistical power to detect subtle differences in miRNA expression and reduces the generalizability of our findings to the broader ARHL population. Although we validated four key DE-miRNAs using RT-qPCR in an additional set of samples (*n* = 3 per group), the overall sample size remains modest, and the results should be interpreted as preliminary. To address the limitation of small sample size, future studies should validate our findings in a larger, independent cohort (e.g., *n* ≥ 50 per group) with diverse demographic backgrounds (including different ages, genders, and regional origins) to improve the generalizability of the results. Additionally, functional experiments (such as in vitro cell models of auditory cell senescence and *in vivo* ARHL animal models) are needed to verify the regulatory roles of the four key DE-miRNAs (hsa-miR-100-5p, hsa-miR-23b-3p, hsa-miR-373-3p, and hsa-miR-27b-3p) in ARHL-related pathways (e.g., ubiquitination, autophagy, and cellular senescence). Longitudinal follow-up studies could further explore the prognostic value of these miRNAs in ARHL progression, providing more robust evidence for their clinical application as diagnostic or prognostic biomarkers.

## 5 Conclusion

The pathogenesis of ARHL is multifaceted. Our results suggest that ubiquitination modification, autophagy process, cellular senescence and nervous system regulation may jointly contribute to the core molecular mechanism of ARHL. The hsa-miR-100-5p, hsa-miR-23b-3p, hsa-miR-373-3p, and hsa-miR-27b-3p may preliminarily act as key regulatory factors to participate in the pathophysiological process of ARHL, providing exploratory evidence for their potential application value as molecular markers. Future validation in larger cohorts and functional studies is required to confirm their clinical utility.

## Data Availability

The RNA-seq data generated in this study have been deposited in the NCBI Sequence Read Archive (SRA) database under the BioProject accession number PRJNA1382517. The data are publicly accessible via the following URL: https://www.ncbi.nlm.nih.gov/sra/PRJNA1382517.
