# Supplementary material for: The differential expression profiles of miRNA in serum-derived exosomes and its potential role in age-related hearing loss
Source: Front Aging Neurosci. 2026 Jan 14;17:1694514. doi: 10.3389/fnagi.2025.1694514 (PMC12847408; doi:10.3389/fnagi.2025.1694514)
Supplement: Supplementary file 1 [file Data_Sheet_1.docx]

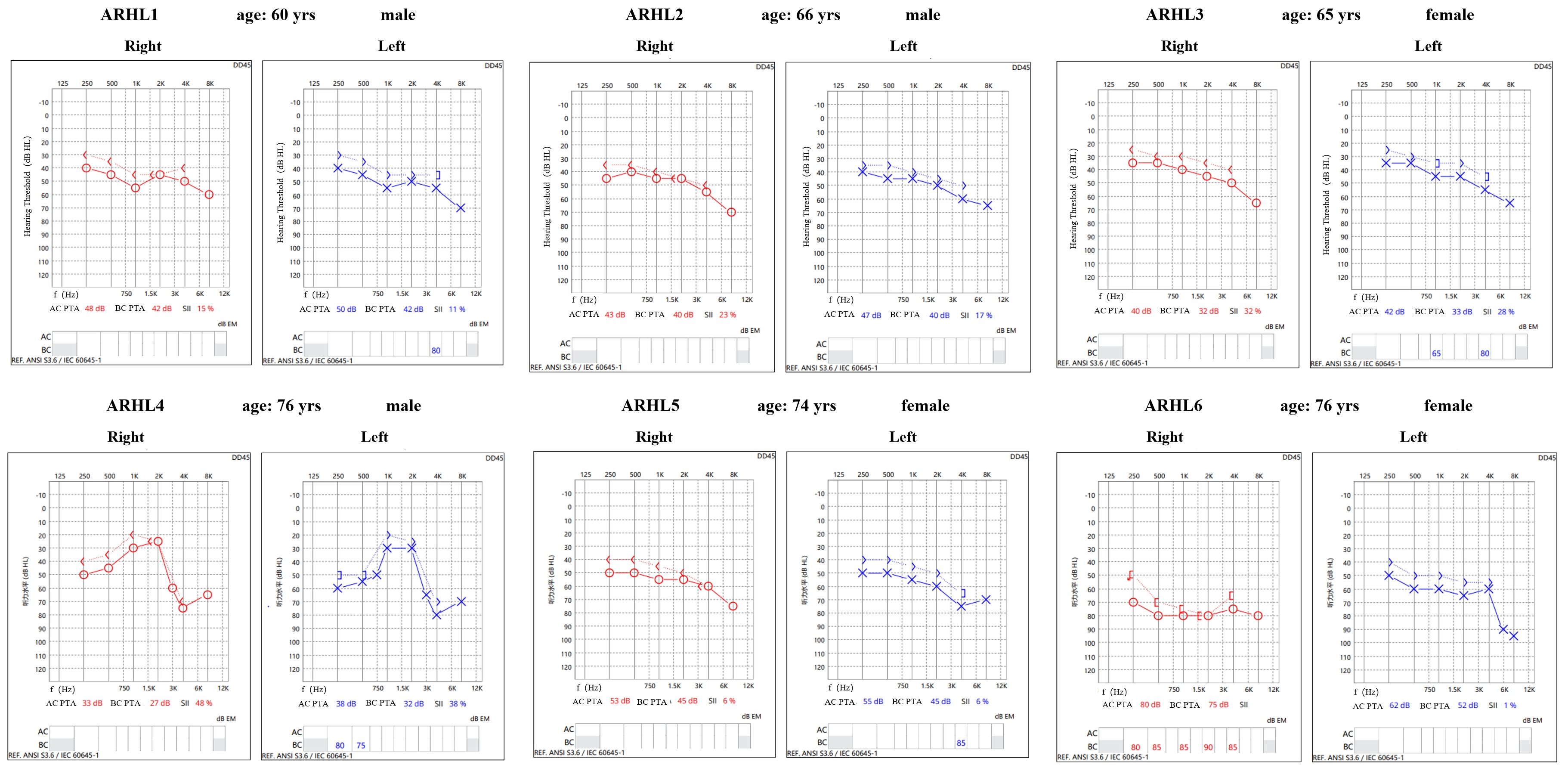


**Supplementary Figure 1** The audiograms of age-related hearing loss (ARHL) cases(n = 6).


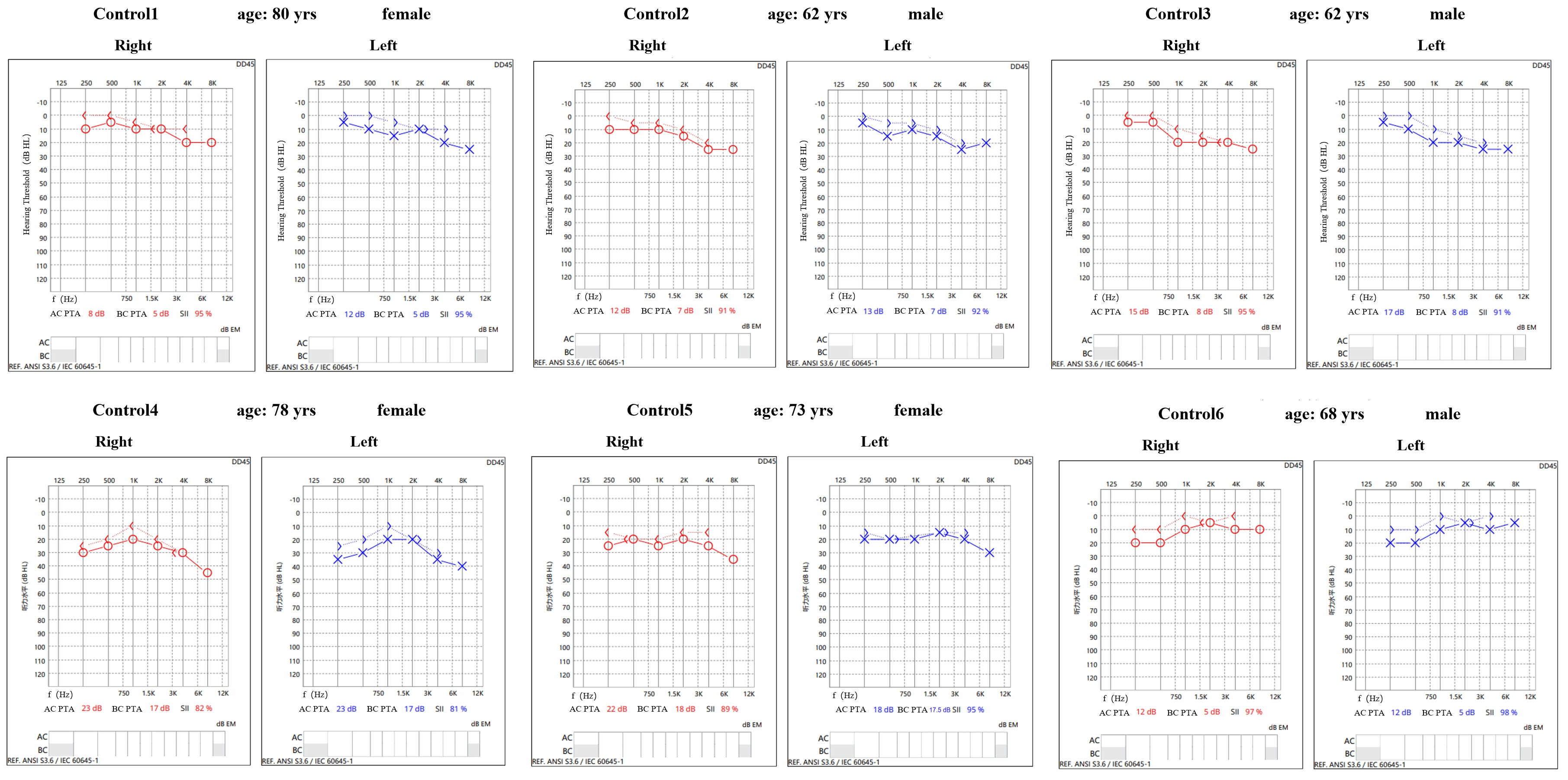


**Supplementary Figure 2** The audiograms of elderly control cases (n = 6).
